# The influence of negative stimulus features on conflict adaption: evidence from fluency of processing

**DOI:** 10.3389/fpsyg.2015.00185

**Published:** 2015-02-26

**Authors:** Julia Fritz, Rico Fischer, Gesine Dreisbach

**Affiliations:** ^1^Department of Experimental Psychology, University of Regensburg, Regensburg, Germany; ^2^Department of Psychology, Technische Universität Dresden, Dresden, Germany

**Keywords:** conflict adaptation, aversive signal, fluency of processing

## Abstract

Cognitive control enables adaptive behavior in a dynamically changing environment. In this context, one prominent adaptation effect is the sequential conflict adjustment, i.e., the observation of reduced response interference on trials following conflict trials. Increasing evidence suggests that such response conflicts are registered as aversive signals. So far, however, the functional role of this aversive signal for conflict adaptation to occur has not been put to test directly. In two experiments, the affective valence of conflict stimuli was manipulated by fluency of processing (stimulus contrast). Experiment 1 used a flanker interference task, Experiment 2 a color-word Stroop task. In both experiments, conflict adaptation effects were only present in fluent, but absent in disfluent trials. Results thus speak against the simple idea that any aversive stimulus feature is suited to promote specific conflict adjustments. Two alternative but not mutually exclusive accounts, namely resource competition and adaptation-by-motivation, will be discussed.

## INTRODUCTION

In an environment full of tempting opportunities and action affordances, appropriate action selection is a constant challenge. For example, grabbing the low fat yogurt instead of the rich but more delicious chocolate mousse from the fridge can be a hard decision. In situations like this, cognitive control supports the selection of the weaker but intended action in the face of a stronger but inadequate action (c.f. Miller and Cohen, 2001). Moreover, when confronted with response conflicts, cognitive control not only enables conflict resolution in the current trial but also adjusts processing parameters such that the cognitive system is better prepared when the response conflict repeats as indicated by reduced response interference in post-conflict trials (Gratton et al., 1992; Botvinick et al., 1999; Notebaert et al., 2001; Stürmer et al., 2002; Caessens et al., 2005; Egner, 2008; Wühr and Kunde, 2008). On a neuronal level, it has been suggested that it is the anterior cingulate cortex (ACC) that detects conflicts and sends this information to the dorsolateral prefrontal cortex which then increases control in the post-conflict trial (e.g., Botvinick et al., 2004; Kerns et al., 2004).

In the past decade, huge advances have been made to further our understanding of the underlying processes that enable such dynamic control adaptations (for recent approaches to conflict adaptation effects see for example Braem et al., 2014; Duthoo et al., 2014; Jiang et al., 2014). Questions of interest concerned the locus and specificity of the adaptation effect (e.g., Kiesel et al., 2006; Kunde and Wühr, 2006; Notebaert and Verguts, 2008; Wendt et al., 2012), the role of episodic retrieval and priming processes (e.g., Mayr et al., 2003; Hommel et al., 2004), the role of learning (e.g., Holroyd and Coles, 2002; Blais et al., 2007; Verguts and Notebaert, 2009; Blais and Verguts, 2012), timing (e.g., Goschke and Dreisbach, 2008; Scherbaum et al., 2011; Pastötter et al., 2013), conflict strength (e.g., Takezawa and Miyatani, 2005; Forster et al., 2011; Wendt et al., 2014), working memory load (Stürmer et al., 2005; Fischer et al., 2010; Soutschek et al., 2012), and context effects in general (e.g., Fischer et al., 2008; Funes et al., 2010).

Only recently, the question of how stress, affect and motivation might influence processing adjustments has moved into the focus of research (e.g., van Steenbergen et al., 2009, 2010, 2012; Kuhbandner and Zehetleitner, 2011; Padmala et al., 2011; Plessow et al., 2011; Stürmer et al., 2011; Braem et al., 2012; see Dreisbach and Fischer, 2012a, for a review). The role of affect in sequential conflict adaptation is of specific interest here due to the increasing evidence that conflicts themselves are experienced as aversive signals (Dreisbach and Fischer, 2012b; Schouppe et al., 2012, 2015; Fritz and Dreisbach, 2013, 2014). For example, presenting Stroop conflict stimuli (Stroop, 1935) as primes eased the evaluation of negative target stimuli and increased the frequency of negative judgments for neutral target stimuli (Dreisbach and Fischer, 2012b; Fritz and Dreisbach, 2013, 2014). Converging evidence in favor of the aversive conflict signal also comes from physiological studies showing increased heart rate (Renaud and Blondin, 1997), larger pupil dilatation (van Steenbergen and Band, 2013; Wendt et al., 2014), and enhanced skin conductance response (Kobayashi et al., 2007) in response to incongruent Stroop stimuli (but see Schacht et al., 2010, who, however, did not use Stroop stimuli but measured physiological activity during a go/no-go paradigm). Given that conflicts are detected by the ACC, and further given that the ACC is also activated by monetary loss (Rainville, 2002), social exclusion (Eisenberger et al., 2003), negative feedback (Nieuwenhuis et al., 2004), and pain (Singer et al., 2004), one might therefore speculate that it is not the response conflict *per se* but the aversive character of the response conflict that triggers the processing adjustments (Botvinick, 2007; Dreisbach and Fischer, in press a, in press b). In fact, Dreisbach and Fischer (2011) found that aversive stimuli can lead to sequential adaptation effects even in the absence of response conflicts. In that study, the authors made use of the fact that fluency of processing, i.e., the experienced ease of stimulus processing, is affectively marked, with low fluency being associated with negative and high fluency with positive affect (Reber et al., 1998; Winkielman et al., 2003)^1^. Dreisbach and Fischer (2011) let participants categorize number words according to magnitude that were either written in an easy (fluent) or hard to read (disfluent) font. In three experiments, the authors found sequential modulations of the fluency effect (performance difference between disfluent and fluent trials) in terms of a smaller fluency effect following disfluent trials. Moreover, van Steenbergen and colleagues repeatedly showed that presenting positive symbols in the inter-trial-intervals of an Eriksen Flanker task (Eriksen and Eriksen, 1974) eliminated conflict adaptation effects (van Steenbergen et al., 2009, 2012; but see Stürmer et al., 2011; Braem et al., 2013b; Notebaert and Braem, in press). Van Steenbergen and colleagues interpreted this result as indication that the positive symbol counteracted the aversive signal of the response conflict and thus eliminated conflict adaptation.

In sum, the observations that (1) aversive signals without response conflict promote sequential processing adjustments (Dreisbach and Fischer, 2011) and (2) positive signals following response conflict eliminate processing adjustments (van Steenbergen et al., 2009, 2012), might suggest that the aversive characteristic of conflicts itself triggers the processing adjustments^2^. One straightforward way to address the question whether it is the aversive conflict signal that triggers conflict adaptation is to increase the aversiveness of a given conflict stimulus and investigate its effects on conflict adaptation. As already mentioned above, perceptual fluency serves as an affective signal with high perceptual fluency being associated with positive affect and low perceptual fluency being associated with negative affect (Reber et al., 1998; Winkielman et al., 2003). Therefore, we manipulated the aversive quality of a conflict signal by presenting classical response interference tasks either with high perceptual fluency (fluent) or with low perceptual fluency (disfluent), expecting that disfluent incongruent trials are more aversive than fluent incongruent trials. Consequently, if it is an unspecific aversiveness conveyed by the conflict that triggers conflict adaptation, we should find increased conflict adaptation on disfluent trials as compared to fluent trials, as disfluency is assumed to increase the general aversiveness of conflicts even further.

## EXPERIMENT 1

### METHOD

#### Participants

Thirty students of the University of Regensburg were tested (23 female; 24 right-handers; mean age: 26.6, SD = 4.1). All participants signed informed consent before the experiment and received 3 Euros or partial course credit after its completion. Data of two participants with RTs that were more than two standard deviations (SDs) above at least one group cell mean were excluded from the analysis, leaving a final sample of 28 participants.

#### Material and procedure

Stimuli consisted of a central color-square horizontally flanked by two color-squares, one on each side. The three horizontally aligned squares subtended a visual angle of 19.9° × 6.6° at a viewing distance of 60 cm. Square colors were red, green, and blue. Congruency was manipulated by color match or mismatch: The color of the central square could either match (congruent stimulus) or mismatch (incongruent stimulus) the color of the two flanking squares which were always of the same color. Fluency was manipulated by figure-ground contrast differences. In fluent stimuli, color saturation was 100%, in disfluent stimuli, color saturation was 50%. Fluency of relevant and irrelevant stimulus dimensions was manipulated to the same extent by stimulus contrast such that no effect on the conflict *per se* is to be expected (Miles and Proctor, 2009). The stimuli were presented centrally on a white background. Participants were instructed to quickly and accurately identify the color of the central square by pressing one of three keys on a QWERTZ keyboard (“c” for green, “v” for red, “b” for blue, respectively) with their index, middle and ring finger of their dominant hand (see Larson et al., 2009, for a similar procedure).

Each trial started with a plus sign as fixation cross for 250 ms, followed by the imperative stimulus that was presented until a response was given. For correct responses, the next trial started after 1000 ms. For errors, the German word for error (Fehler) appeared and remained on screen for 1000 ms. After an additional 500 ms, the next trial started. The experiment started with a short test to exclude color blindness, followed by a color-to-key-mapping practice block of 12 randomly presented imperative stimuli and a second practice block of 24 imperative stimuli where participants were introduced to the fluency manipulation. After that, one practice block of 120 trials followed consisting of 30 congruent and 30 incongruent fluent and disfluent trials, respectively. This practice block was followed by three experimental blocks of 120 trials each. Blocks were separated from one another by self-paced breaks. Repetition of identical target stimuli was not allowed. Because we were interested in how fluency modulates conflict adaptation, we presented fluent and disfluent trials in runs of 10 in a given block while for the assessment of conflict adaptation, conflict vs. non-conflict trials varied randomly from trial to trial. The experiment lasted about 25 min.

#### Design

A 2 (Congruency_*N*_) × 2 (Congruency_*N-1*_) × 2 (Fluency) repeated measures design was used.

#### Data preprocessing

We excluded the first two trials of each fluency block of 10 trials length in order to remove possible transition effects from the previous fluency condition. In order to decrease the influence of low-level feature repetitions and to maximize cognitive control involvement in conflict adaptation (e.g., Egner, 2007), partial priming trials [whenever the color of either the central or flanking stimulus repeated from trial N-1 to trial N (42.3%)] and negative priming trials [whenever the color of the flanking squares in trial N-1 was the color of the central square in trial N (18.5%)] were excluded prior to analysis (see also Ullsperger et al., 2005; Bugg, 2008; Larson et al., 2009; Wendt et al., 2014)^3^. For error data analysis, mean error rates for the remaining data (on average 134 trials per participant) were computed for each cell of the 2 (Congruency_*N*_) × 2 (Congruency_*N-1*_) × 2 (Fluency) design and entered into a repeated measures analysis of variance (ANOVA). Additionally, for RT data analysis, erroneous as well as post-error trials (together 6.7%) and all RTs that exceeded more than two SDs from the individual cell mean (4.9%) were excluded prior to analysis. For the remaining data (on average 121 trials per participant), mean RTs for each cell of the 2 (Congruency_*N*_) × 2 (Congruency_*N-1*_) × 2 (Fluency) design were computed and a repeated measures ANOVA was conducted.

### RESULTS AND DISCUSSION

#### Error data

**FIGURE 1:**
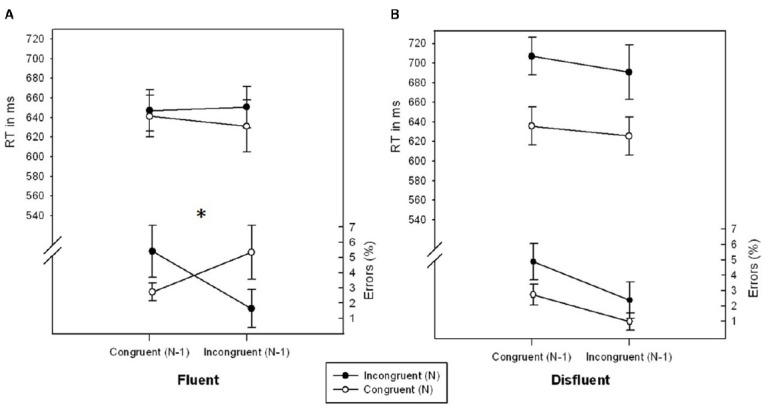
**RTs (ms) and error rates (%) as a function of Congruency_*N*_ and Congruency_*N-1*_ for fluent (A) and disfluent (B) trials of Experiment 1.** Error bars represent standard errors of the mean. The symbol “^*^” denotes a significant interaction Congruency_*N*_ × Congruency_*N-1*_.

There was a marginally significant interaction of Congruency_*N*_ × Congruency_*N-1*_, *F*(1,27) = 3.375, *p* = 0.077, η^2^ = 0.111, reflecting a typical conflict adaptation effect. The congruency effect was less pronounced following incongruent_*N-1*_ (–1.16%) as compared to following congruent_*N-1*_ trials (2.42%). Importantly, this conflict adaptation effect was further modulated by Fluency, *F*(1,27) = 4.675, *p* < 0.05, η^2^ = 0.145 (see Figure 1). In fluent trials, participants showed a significant conflict adaptation effect, *F*(1,27) = 5.783, *p* < 0.05, η^2^ = 0.176, i.e., an inverted congruency effect for trials following incongruent trials (–3.67%) as compared to following congruent trials (2.69%). In disfluent trials, however, the effect of Congruency_*N*_ was unaffected by Congruency_*N-1*_, *F* < 1, *p* > 0.706, η^2^ < 0.006. No further effects were significant, all *F*s < 2.873, all *p*s > 0.101, all η^2^s < 0.097.

#### RT data

The main effects of Congruency_*N*_, *F*(1,27) = 22.515, *p* < 0.001, η^2^ = 0.464, and Fluency, *F*(1,27) = 5.051, *p* < 0.05, η^2^ = 0.163, were significant. RT was lower for congruent_*N*_ (633.45 ms) as compared to incongruent_*N*_ (673.90 ms) trials and lower for fluent (642.59 ms) as compared to disfluent (664.76 ms) trials. Furthermore, there was a significant interaction of Congruency_*N*_ × Fluency, *F*(1,27) = 7.239, *p* < 0.05, η^2^ = 0.218: The Congruency effect was less pronounced in fluent (12.63 ms) as compared to disfluent trials (68.27 ms). No further effects were significant, all *F*s < 1.019, all *p*s > 0.321, all η^2^s < 0.039.

The main results of Experiment 1 can be summarized as follows: The higher order interaction of Congruency_*N*_ × Congruency_*N-1*_ × Fluency found in the error data showed the usual significant conflict adaptation effect on fluent trials (i.e., stimuli with high stimulus contrast as used in standard paradigms), and a significantly reduced and virtually absent conflict adaptation effect for disfluent trials. The results thus suggest that, if anything, increasing the general aversiveness of conflicts by reducing the stimulus contrast *eliminates* the conflict adaptation effect. This contradicts the idea that adding unspecific aversiveness to a conflict stimulus increases specific adaptation effects. In order to consolidate the findings from Experiment 1, we ran a second experiment with a different response conflict paradigm, i.e., a manual version of the Stroop task (Stroop, 1935). If the results from Experiment 1 (the modulation of the conflict adaptation effect by fluency with an elimination thereof in disfluent trials) can be replicated in Experiment 2, it can be ruled out that the effects were driven by paradigm specific parameters and thus highlight the findings’ generalizability.

## EXPERIMENT 2

### METHOD

#### Participants

Thirty students of the University of Regensburg were tested (23 female; 28 right-handers; mean age: 23.1, SD = 3.7). All participants signed informed consent before the experiment and received 3 Euros or partial course credit after its completion. Data of three participants with RTs that were more than two SDs above at least one group cell mean were excluded from the analysis, leaving a final sample of 27 participants.

#### Material and procedure

Stimuli were the German color words for RED (rot), GREEN (grün), and BLUE (blau) printed in red, green, and blue (RGB values of 255,0,0; 0,255,0; and 0,0,255, respectively). The words were written in Arial bold, 24 point, each letter subtending a visual angle of approximately 0.8° × 0.8° at a viewing distance of 60 cm. Congruency was manipulated by color-word match or mismatch: The print color could either match (congruent stimuli) or mismatch (incongruent stimuli) the word meaning of the stimulus. Again, fluency was manipulated by figure-ground contrast differences. In fluent stimuli, color saturation was 100%, in disfluent stimuli, color saturation was 50%. The stimuli were presented centrally on a white background. Participants’ task was to quickly and accurately identify the print color of the word while ignoring its meaning by pressing one of three keys on a QWERTZ keyboard (“c” for green, “v” for red, “b” for blue, respectively) with their index, middle, and ring finger of their dominant hand. Trial and block procedure remained the same as in Experiment 1.

#### Design

A 2 (Congruency_*N*_) × 2 (Congruency_*N-1*_) × 2 (Fluency) repeated measures design was used.

#### Data preprocessing

We excluded the first two trials of each fluency block of 10 trials length in order to remove possible transition effects from the previous fluency condition. Furthermore, partial priming trials [whenever the color or color word repeated from trial N-1 to trial N (46.6%)] and negative priming trials [whenever the color word in trial N-1 was the color of the stimulus in trial N (16.2%)] were excluded prior to analysis to ensure that priming effects did not mask conflict adaptation. For error data analysis, mean error rates for the remaining data (on average 130 trials per participant) were computed for each cell of the 2 (Congruency_*N*_) × 2 (Congruency_*N-1*_) × 2 (Fluency) design and entered into a repeated measures ANOVA. Additionally, for RT data analysis, erroneous as well as post-error trials (together 7.5%) and all RTs that exceeded more than two SDs from the individual cell mean (4.6%) were excluded prior to analysis. For the remaining data (on average 118 trials per participant), mean RTs for each cell of the 2 (Congruency_*N*_) × 2 (Congruency_*N-1*_) × 2 (Fluency) design were computed and a repeated measures ANOVA was conducted.

### RESULTS AND DISCUSSION

#### Error data

There was a significant main effect of Congruency_*N*_, *F*(1,26) = 16.335, *p* < 0.001, η^2^ = 0.386, and a marginally significant effect of Fluency, *F*(1,26) = 3.028, *p* = 0.094, η^2^ = 0.104. Error rates were lower for congruent_*N*_ (2.39%) as compared to incongruent_*N*_ (4.65%) trials and lower for fluent (3.19%) as compared to disfluent trials (3.85%). No further effects were significant, all *F*s < 2.331, all *p*s > 0.138, all η^2^s < 0.083.

#### RT data

**FIGURE 2:**
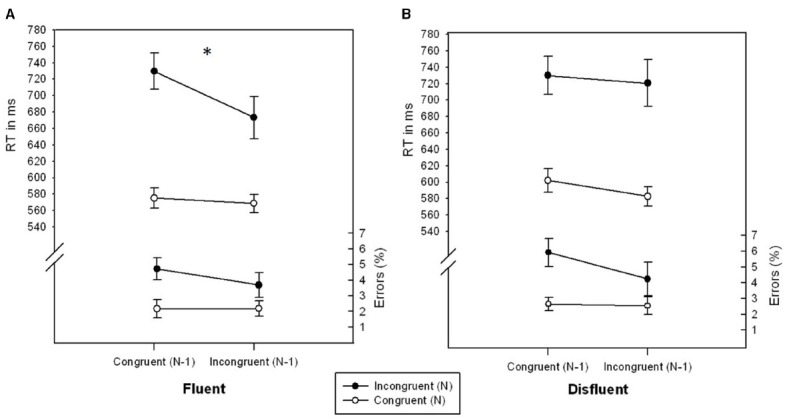
**RTs (ms) and error rates (%) as a function of Congruency_*N*_ and Congruency_*N-1*_ for fluent (A) and disfluent (B) trials of Experiment 2.** Error bars represent standard errors of the mean. The symbol “^*^” denotes a significant interaction Congruency_*N*_ × Congruency_*N-1*_.

The main effects of Congruency_*N*_, *F*(1,26) = 86.941, *p* < 0.001, η^2^ = 0.770, Congruency_*N-1*_, *F*(1,26) = 10.821, *p* < 0.01, η^2^ = 0.294, and Fluency, *F*(1,26) = 5.371, *p* < 0.05, η^2^ = 0.171, were significant. RT was lower for trials following incongruent trials (636.34 ms) as compared to congruent trials (659.30 ms), lower for congruent_*N*_ (582.21 ms) as compared to incongruent_*N*_ (713.42 ms) trials and lower for fluent (636.71 ms) as compared to disfluent (658.92 ms) trials. As in the accuracy data of Experiment 1, the interaction of Congruency_*N*_ × Congruency_*N-1*_ × Fluency was significant, *F*(1,26) = 6.604, *p* < 0.05, η^2^ = 0.203 (see Figure 2). In fluent trials, participants showed a significant conflict adaptation effect, *F*(1,26) = 8.220, *p* < 0.01, η^2^ = 0.240, i.e., a smaller congruency effect for trials following incongruent trials (104.56 ms) as compared to following congruent trials (154.42 ms). In disfluent trials, however, the congruency effect was unaffected by Congruency_*N-1*_ (137.98 ms following incongruent and 127.86 ms following congruent trials), *F* = 0.323, *p* = 0.574, η^2^ = 0.012. No further effects were significant, all *F*s < 1.932, *p*s > 0.175, all η^2^s < 0.070.

In Experiment 2, the higher order interaction Congruency_*N*_ × Congruency_*N-1*_ × Fluency was significant in the RT data. Again, the conflict adaptation effect was only significant in fluent trials but was eliminated in disfluent trials. Taken together, both experiments brought up converging evidence that sequential conflict adaptation, if present in fluent trials, is entirely reduced in disfluent trials. This contradicts the idea that any aversive signal is suited to trigger stimulus-specific adaptation effects.

## GENERAL DISCUSSION

Based on a theoretical framework of ACC functioning (Botvinick, 2007) and recent findings of (1) conflict aversiveness (Dreisbach and Fischer, 2012b; Schouppe et al., 2012, 2015; Fritz and Dreisbach, 2013, 2014), (2) elimination of conflict adaptation by positive action effects (van Steenbergen et al., 2009, 2012), and (3) sequential adaptation triggered by non-conflict aversive (disfluent) stimuli (Dreisbach and Fischer, 2011), we directly tested whether increasing the aversive value of conflict stimuli also increases sequential adaptation effects. To this end, we presented conflict stimuli with either high or low perceptual fluency. Because disfluency is experienced as aversive signal (Reber et al., 1998), this manipulation is suited to modulate the affective valence of conflict stimuli. If conflict adaptation is triggered by the aversive nature of conflict stimuli independently from the conflict information, then the increased negative valence of disfluent incongruent as compared to fluent incongruent trials might increase adaptation effects.

Results from both experiments, however, did not support this idea. In contrast, whenever the typical conflict adaptation was found for fluent trials (in the error data in Experiment 1 and in the RT data in Experiment 2), disfluency eliminated conflict adaptation effects entirely. And this cannot be explained by reduced conflict strength on disfluent trials because conflict was either unaffected by the fluency manipulation (Experiment 2) or even increased (Experiment 1) for disfluent trials^4^.

In both experiments, conflict adaptation was only present in one of the dependent measures, i.e., error rates in Experiment 1 and RT data in Experiment 2. Thus, neither RT data in Experiment 1 nor error rates in Experiment 2 were further modulated by disfluency. Indeed, there have been many studies reporting similar findings, i.e., conflict adaptation effects being only present in RT data OR error data (see, e.g., Ullsperger et al., 2005; Bugg, 2008; van Steenbergen et al., 2010, 2012; Puccioni and Vallesi, 2012; Soutschek et al., 2012). So far, there has been no study that directly addressed why the conflict adaptation effect is sometimes found in the RT data while it is found in the error data in other cases. The important result of our study, however, is that the dependent measure that showed the typical conflict adaptation effect on fluent trials in the respective experiment (i.e., error rates in Experiment 1 and RT data in Experiment 2) also brought up a higher order interaction with fluency: While conflict adaptation is intact on fluent trials, disfluency leads to its elimination.

These consistent findings from two independent experiments have an important implication: They demonstrate that increasing unspecific aversiveness, for example by decreasing fluency of processing, does not inevitably lead to stronger conflict adaptation but in contrast may even diminish it. Thus, it is conceivable that aversiveness might need to be tied to conflict processing and not to stimulus processing in general.

An important question, however, remains: why does reduced fluency of processing (and thus, increased aversiveness) not only *not* increase or not affect conflict adaptation, but eliminates it? Here, a potential answer could be that reducing fluency of processing might come with side effects other than the aversive connotation that could directly have affected conflict adaptation.

For example, processing of disfluent stimuli might have increased processing demands and invested effort (e.g., Dreisbach and Fischer, 2011). There is already ample evidence that conflict adaptation is modulated by processing demands of primary task processing. For example, Fischer et al. (2008) had participants complete a number magnitude task (i.e., indicate whether a given number was bigger or smaller than 5) combined with a Simon task (i.e., numbers appeared on the right or left side of the screen). They found the typical sequential conflict adaptation in the Simon task which was further modulated by the cognitive demand of the number magnitude task: Following numbers close to the reference standard (high processing demand), the Simon adaptation was smaller than following numbers far from the reference standard (low processing demand). Likewise, Soutschek et al. (2012) reported evidence that high working memory load eliminates conflict adaptation in the Stroop task. Applied to our results presented here, one might thus argue that disfluent trials draw on processing resources that were then not available for conflict adaptation. Does that imply that the aversive character of disfluency had no effect in our study? Interestingly, Pessoa (2009) claimed that not only different cognitive processes share and compete for the same restricted resource capacities, but that cognitive and affective processes do so as well. Indeed, it has been shown that performance in incongruent trials decreased when preceded by an affective task-irrelevant picture (Hart et al., 2010), suggesting that the processing of the affective stimulus consumed resources that would otherwise have benefited conflict resolution. In the same line and more directly related to our study, Padmala et al. (2011) reported that presenting highly arousing negative pictures as compared to neutral pictures in inter-trial-intervals of a Stroop-like word-face task eliminated conflict adaptation effects (see also Braem et al., 2013b). The authors, too, explained this finding in terms of resource competition: The resources that are necessary for post-conflict adaptation were consumed by the processing of the arousing aversive pictures and were then lacking for conflict adaptation. In the light of these findings, the resource competition account might explain our data, as well. As disfluency is associated with negative affect, the processing of the aversive quality in disfluent blocks may have demanded resources that would otherwise have been used to adapt control in post-conflict trials^5^.

A second, very recent line of research that is of interest to our results deals with the motivational impact of conflict resolution on conflict adaptation. According to this adaptation-by-motivation account, conflict adaptation is triggered by the rewarding experience of conflict resolution (Braem et al., 2012). This idea is grounded on the observation that solving a difficult task is more rewarding than solving an easy task (Shalley and Oldham, 1985; Satterthwaite et al., 2012). For example, Satterthwaite et al. (2012) used an n-back task and were able to show that the activation in the ventral striatum, a key region of dopamine production, increased with increasing task difficulty. The most direct evidence for the role of reward for conflict adaptation has recently been put forward by Braem et al. (2012). In that study, participants were presented with an Eriksen Flanker task (Experiment 1). In the experimental condition, 25% of trials of a given block were rewarded for correct and fast performance, whereas in the remaining 75% of the trials, no reward was given. In the control condition, no reward was given ever. Results brought up sequential conflict adaptation effects in the control condition (no reward) and in rewarded trials in the experimental condition. Intriguingly, no such conflict adaptation was found for unrewarded trials in the reward context. According to the authors, the extrinsic reward signal on 25% of trials replaced or overshadowed the intrinsic reward signal normally generated in standard (no-reward) conflict tasks. As a consequence, the no-reward trials lacked the intrinsic rewarding experience that would have been necessary to trigger conflict adaptation.

Back to the data presented here, the adaptation-by-motivation account also fits with our findings. Notably, fluency of processing has been shown to modulate motivation directly. For example, Song and Schwarz (2008) found that participants were less motivated to carry out a task that was described in a hard to read (disfluent) font as compared to a task described in an easy to read (fluent) font. Applied to our experiments, the continuous experience of disfluency throughout the mini-blocks of disfluent trials might have reduced the motivation to adapt. Put differently, in disfluent mini-blocks, the rewarding effect of a successful conflict resolution might have been counteracted by the discouraging continuous disfluent experience. Therefore, the repeated disfluent experience eliminated the intrinsic reward signal that typically follows successful conflict resolution, thereby decreasing the conflict adaptation effect. Further support for this motivational account comes from studies showing that an increase in participants’ motivation goes along with decreased RTs and error rates and decreased congruency effects, mimicking our results in the fluent as compared to the disfluent conditions (e.g., Veling and Aarts, 2010; Padmala and Pessoa, 2011; Soutschek et al., 2014).

In sum, the two accounts presented above, i.e., the adaptation-by-motivation account, and the resource competition account, are equally well suited to explain our results. In fact, they are not mutually exclusive but closely intervened. After all, the negative valence of disfluency (just as the negative valence of conflict stimuli, see Botvinick, 2007) might at least in part be due to the increased processing demands of disfluent (and incongruent) trials. The only caveat might be that our results are hard to reconcile with the interpretation of van Steenbergen et al. (2009) outlined in the Introduction. To reiterate, the authors found no conflict adaptation following positive signals and argued that the positive signals presumably counteracted the aversive character of the conflict stimulus. Alternatively, and in line with the adaptation-by-motivation account, the positive signals in the van Steenbergen study that were presented as non-contingent performance feedback might have signaled that successful performance is not a value by itself and thereby counteracted the intrinsic reward signal (see also Dreisbach and Fischer, 2012a, for a more thorough discussion). In sum, random reward (van Steenbergen et al., 2009, 2012; see also Stürmer et al., 2011), no-reward in a reward context (Braem et al., 2012) and repeated experience of disfluency (the results presented here) have all been found to reduce or eliminate conflict adaptation. The common underlying mechanism might be that in all these situations, the intrinsic reward signal after successful conflict resolution was reduced.

It is important to note that the present findings and the suggested interpretations do not at all contradict the repeatedly shown aversive nature of conflict stimuli and their role for conflict adaptation (Dreisbach and Fischer, 2012b; Schouppe et al., 2012, 2015; Fritz and Dreisbach, 2013, 2014). What we have shown here is that increasing the negative valence of conflict stimuli via disfluency (and thus independently from conflict strength) does not increase conflict adaptation effects. But at the same time, it is well documented that (1) disfluency triggers processing adjustments in terms of a reduced fluency effect (Dreisbach and Fischer, 2011) and that (2) conflict adaptation effects increase with increasing conflict strength (Takezawa and Miyatani, 2005; Forster et al., 2011; Wendt et al., 2014). Therefore, we argue that the aversive signal conveyed by the amount of conflict triggers conflict adaptation. Yet it seems that aversive stimulus information from different sources (here: from perceptual fluency vs. response conflict) does not add up to increase sequential conflict adaptation. That is, the aversiveness must be tied to conflict processing and not to stimulus processing in general.

### Conflict of Interest Statement

The authors declare that the research was conducted in the absence of any commercial or financial relationships that could be construed as a potential conflict of interest.

## Footnotes

1 It is important to note that it has been shown empirically that not only is perceptual fluency associated with positive affect but also that perceptual disfluency is associated with negative affect. In three experiments, Reber et al. (1998) applied different manipulations of perceptual fluency and examined the consequences on preference judgments. In Experiment 1, participants judged drawings that were preceded by mismatching primes (i.e., perceptually disfluent stimuli) as being less pretty than drawings that were preceded by matching primes. In Experiment 3, participants stated to dislike stimuli more that were presented for shorter periods of time than stimuli that were presented for longer time periods. Finally, and most similar to the manipulation of perceptual fluency used in the present study, in Experiment 2, Reber et al. (1998) let participants judge circle stimuli that varied in figure-ground contrast. They found that the circles were judged as less pretty and more ugly with decreasing figure-ground contrast, i.e., with decreasing perceptual fluency.

2 There are a couple of studies, however, suggesting that it is not the affective value of conflicts but the *rewarding* effect of conflict *resolution* that leads to post-conflict adjustment (Braem et al., 2012; see also Schouppe et al., 2015).

3 Because the *post hoc* removal of all stimulus/feature repetitions is standard procedure in studying conflict adaptation, the experiment was from the beginning designed to provide a sufficiently large number of trials per cell. An a priory exclusion of critical stimulus sequences was not considered an option as it induces expectation biases. For a recent discussion of theoretical and practical guidelines in the investigations of conflict adaptation see Duthoo et al. (2014) and Egner (2014).

4 Miles and Proctor (2009) found that decreasing both, the discriminability of the relevant and irrelevant stimulus feature, does not change the magnitude of the congruency effect in two response conflict paradigms. Thus, we assumed that in our experiments, the fluency manipulation would likewise leave the congruency effect unaffected. However, the interaction of fluency with congruency was significant for the Flanker task in Experiment 1. Although we do not have an explanation for this effect, still, this does not weaken our argument: conflict adaptation is absent in disfluent as compared to fluent trials *even though* conflict magnitude was bigger in disfluent trials. It seems thus that the demotivating effect of continued disfluency (see Discussion below) overrules the effect of enhanced conflict aversiveness.

5 An alternative, yet similar, explanation of reduced conflict adaptation effects in affectively negative conditions grounds on the idea that the effects of negative mood (cf. van Steenbergen, 2015) or more generally, arousal (cf. Braem et al., 2013a), on cognitive control follow an inverted U shaped function: while moderate levels of arousal are beneficial for cognitive control processes, too little or too much arousal is detrimental. For example, Braem et al. (2013b) found that the influence of punishment on conflict adaptation critically depends on individual punishment sensitivity: while punishing individuals low in punishment sensitivity [as measured with the Behavioral Inhibition System (BIS) scale] increased conflict adaptation in post-punishment trials, punishing highly punishment sensitive participants did not modulate conflict adaptation but lead to a general slow-down in RTs in post-punishment trials. The authors interpret their data in the framework of the Yerkes and Dodson (1908) law and suggest that for highly punishment sensitive individuals, punishment-associated arousal was too high to benefit conflict adaptation. However, we do not think that this framework is suited to thoroughly explain our results, given that disfluency can hardly be compared to the aversive experience of punishment.
